# Consistency and Reliability Analyses of a Comprehensive Index for the Evaluation of Teeth Alignment Performance

**DOI:** 10.3390/jcm11041016

**Published:** 2022-02-16

**Authors:** Andrea Mapelli, Marco Serafin, Carolina Dolci, Daniele Gibelli, Alberto Caprioglio, Chiarella Sforza, Gianluca Martino Tartaglia

**Affiliations:** 1 Department of Biomedical, Surgical and Dental Sciences, University of Milan, 20122 Milan, Italy; andrea.mapelli@unimi.it (A.M.); carolina.dolci@unimi.it (C.D.); alberto.caprioglio@unimi.it (A.C.); gianluca.tartaglia@unimi.it (G.M.T.); 2 Department of Biomedical Sciences for Health, University of Milan, 20133 Milan, Italy; daniele.gibelli@unimi.it (D.G.); chiarella.sforza@unimi.it (C.S.); 3 Fondazione IRCCS Cà Granda, Ospedale Maggiore Policlinico, 20122 Milan, Italy

**Keywords:** 3D-printed aligners, clear aligners, digital orthodontics, performance index, teeth movement

## Abstract

(1) Introduction: The purpose of this work was to describe a method and propose a novel accuracy index to assess orthodontic alignment performance. (2) Methods: Fifteen patients who underwent orthodontic treatment using directly printed clear aligners were recruited. The study sample included 12 maxillary and 10 mandibular arches, whose pre-treatment, predicted and post-treatment digital models were superimposed on the untreated posterior teeth by means of a best-fit surface-based registration, which was also used to transfer three anatomical landmarks, digitally labeled on the crown of each anterior moving tooth, from the pre-treatment to the predicted and post-treatment models. The Teeth Alignment Performance (TAP) index, quantifying how close the final landmarks were to their expected final position, was proposed as an accuracy index of both individual tooth and group of teeth movement, and its inter-examiner repeatability was tested. (3) Results: No systematic inter-rater discrepancy associated with TAP was observed (*p* > 0.05), not even when a slight systematic inter-rater difference in landmark labelling was detected (for the upper central incisors, *p* < 0.001). In addition, all Intra-class Correlation Coefficient (ICC) values showed excellent inter-rater agreement (>0.95), and the small Random Error of Measurement (REM), ranging from 1% for the arch TAP to 3% for the lower canine TAP, indicated that this accuracy index is highly repeatable. (4) Conclusions: The TAP index was proven to be comprehensive, consistent and reliable in assessing the performance of teeth alignment according to a digital plan. The proposed method is also suitable to be implemented in the clinical digital workflow.

## 1. Introduction

Three-dimensional (3D) digital dental models and procedures have become widely used in orthodontics. Current scanning systems, both indirect-desktop and direct-intraoral types, virtually reproduce accurate and reliable copies of the dental arches [1]. In addition, recent advances in software and manufacturing capabilities have made it possible to build both customized orthodontic arch-wires and clear aligners, based on a virtual setup of the dentition [2].

To support orthodontists evaluating a malocclusion in terms of treatment need, complexity, or outcome, several occlusal indices have been developed over the years. Among the most widely used are the American Board of Orthodontics Objective Grading System (ABO-OGS) score, the Peer Assessment Rating (PAR) score, the Index of Complexity Outcome and Need (ICON) and the Dental Aesthetic Index (DAI) [3]. These indices evaluate both quantitative and qualitative occlusal features, the latter of which partially reduce the objectivity of the measurements; these features are then differently weighted and summed to provide a final score, according to elaborate protocols [4,5]. On the other side, some researchers [6,7] still use the Irregularity Index [8], a simpler technique that measures the occlusal projection of linear distances between anatomical contact points of adjacent teeth, though its use has been questioned in terms of effectiveness [9], accuracy [10], and repeatability [11].

In addition, today it is possible to quantify treatment changes and discrepancies between the expected and the actual treatment results through mathematical superimposition of the digital pre-treatment model, the predicted model, and the achieved post-treatment one. Each tooth movement can be analyzed in its six spatial Degrees of Freedom (6 DOF), which include three translations along the reference axes (commonly mesial-distal, buccal-lingual, and occlusal-gingival) and three rotations around the same axes (torque, tip, and rotation) [12]. As well as the absolute measures, percentage values, showing how closely the final predicted position and orientation have been achieved at the end of a treatment, can be reported for all the six movement components of each tooth [13,14]. However, the calculation of these accuracy indices loses validity in certain circumstances, such as when they provide negative or inconsistent values. In addition, different dimensions, such as distances and angles, cannot be directly pooled together to obtain a proper, comprehensive index of treatment accuracy.

The aim of this study was to develop a quantitative index to assess the performance of an orthodontic treatment according to its digital plan; the index should be comprehensive, consistent in all conditions, and useful both for a clinician and a researcher. Consistency and reliability of the index were checked when maxillary and mandibular anterior teeth movement was sought.

## 2. Materials and Methods

### 2.1. Subjects

Maxillary and/or mandibular dental arches of 15 healthy patients (14 females, 1 male, age range 13–72 years, mean age 34 years), who underwent single- (8 subjects) or dual-arch (7 subjects) orthodontic treatment, were retrospectively collected for the study. All the selected patients were consecutively treated using a new generation of directly printed clear aligners (DonatelloSmile, 3D Objects & Data Software SA, Taverne, Switzerland) from February 2020 to January 2021.

Selection was based on the following inclusion criteria: available pre-treatment and post-treatment records; treatment plan digitally set up and involving the six upper and/or lower front teeth (canine to canine) exclusively; no auxiliary appliances other than composite attachments; full permanent dentition except third molars. Exclusion criteria were as follows: partially erupted teeth, poor quality of pre-treatment or post-treatment records, or dental restorations during the treatment.

No specific malocclusion classes were selected, in order to test the method in a wide range of different conditions (Class I, 11 patients; Class II, 3 patients; Class III, 1 patient). Interproximal enamel reduction (IPR) was applied in 8 patients, with a maximum local enamel removal of 0.2 mm.

The main orthodontic treatment goal, agreed with the patients, was not to achieve a therapeutic optimum, but to improve each patient’s appearance in the anterior region by resolving anterior misalignment, crowding, or gaps.

Patients were instructed to wear each aligner at least 21 h a day, except for during meals and oral hygiene procedures, and to replace aligners with the next ones weekly. The treatment duration ranged from 5 weeks to 9 months.

The present retrospective study was conducted in accordance with the Declaration of Helsinki and approved by the Ethics Committee of University of Milan (protocol code IRB 05/2019 doc SO 03). All the analyzed individuals gave their written informed consent to the orthodontic treatment and the anonymous processing of their data.

### 2.2. Data Collection

The sample included a total of 22 individual digital arches (12 maxillary, 10 mandibular), each collected both before and at the end of the treatment (end of the first session when refinement was needed). Before beginning the treatment, all patients underwent either a two-phase polyvinyl siloxane (PVS) impression, then obtaining plaster casts (9 arches), or an intraoral scan (Trios, 3Shape, Copenhagen, Denmark) (13 arches). At the end of the treatment, 6 arches were recorded with the same intraoral scanner, while 16 arches were obtained via PVS impressions. To obtain the relative digital models, the 25 plaster casts were digitized by means of a desktop scanner (Deluxe, Open Tech 3D Srl, Brescia, Italy).

All treatments were digitally planned, and the final predicted models (22 arches) exported through Maestro Dental Studio CAD software (Age Solutions Srl, Pontedera, Italy), together with the indication of the target (moved) teeth (overall, 83 teeth).

### 2.3. Data Analysis

**Figure 1:**
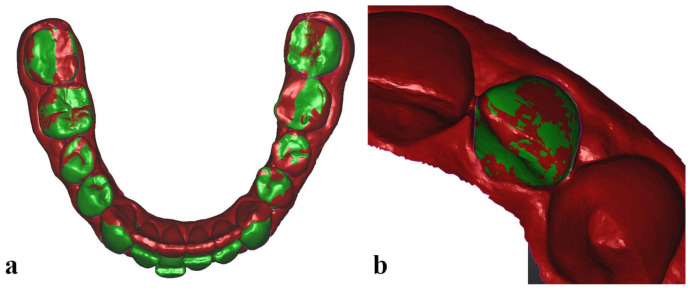
Superimposition of two dental arches (segmented pre-treatment in green, unsegmented post-treatment in red) using the untreated posterior teeth as surface matching reference (**a**), and superimposition of a segmented target tooth of the pre-treatment model (green) over the equivalent tooth of the unsegmented post-treatment arch (red) (**b**), using a surface-based marker-less registration.

All pre-treatment (22 initial, Mi) and predicted (22 expected, Ex) digital models, with their crowns previously segmented in the CAD software, together with post-treatment (22 final, Mf) digital models were uploaded in Optical RevEng software (Open Tech 3D Srl, Brescia, Italy) for the analysis protocol. The 3 corresponding dental arches were first superimposed by means of a best-fit surface-based registration, a mathematical method that minimizes the distance between two separate surfaces [12], using the untreated posterior teeth (all molars and second premolars) as surface matching reference (Figure 1a).

**Figure 2:**
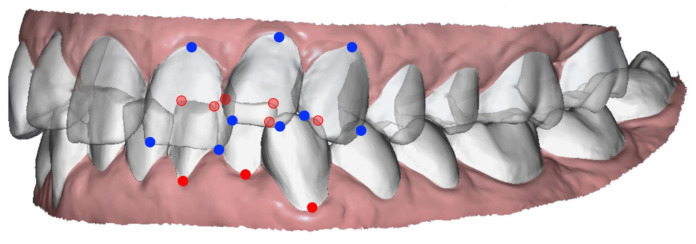
Anterior teeth landmarks in the maxillary (blue circles) and mandibular (red circles) arches. Only landmarks of the left side are shown.

Then, two orthodontists (the authors C.D. and M.S.), previously trained to use the software, independently proceeded with the analysis protocol. For each of the 22 initial Mi models, 3 landmarks were labelled on each segmented target tooth: mesial and distal points of the incisal edge (the canine ridge for the canines) and the gingival limit of the buccal FACC (Facial Axis of the Clinical Crown) (Figure 2).

Then, all the target teeth of a Mi model, together with their 3 landmarks, were superimposed over the equivalent teeth of the corresponding Ex and Mf models, using a surface-based marker-less registration (Figure 1b). The three-dimensional coordinates (x,y,z) of the target teeth landmarks of each model were recorded and imported into a dedicated Excel spreadsheet (Microsoft Excel, Microsoft, Redmond, WA, USA).

For each target tooth, a Tooth Dimensional Indicator (TDI) was calculated as the sum of the three linear distances connecting the three landmarks.

For each landmark *i* of a tooth: the linear distances connecting the landmark *i* in the Ex model with the same landmark *i* in the Mi and Mf models represent its predicted displacement and its missed displacement, respectively. The Teeth Alignment Performance index (TAP) was then determined by the following equation:

$$\mathrm{TAP}=\left[1-\frac{\Sigma \left({\mathrm{Mf}}_{i}-{\mathrm{Ex}}_{i}\right)}{\Sigma \left({\mathrm{Mi}}_{i}-{\mathrm{Ex}}_{i}\right)}\right]\%$$

and could be calculated both for each individual target tooth (when the sum of its 3 landmark displacements is considered) and for the entire arch (when the 3 landmark displacements of all the target teeth are summed together).

**Figure 3:**
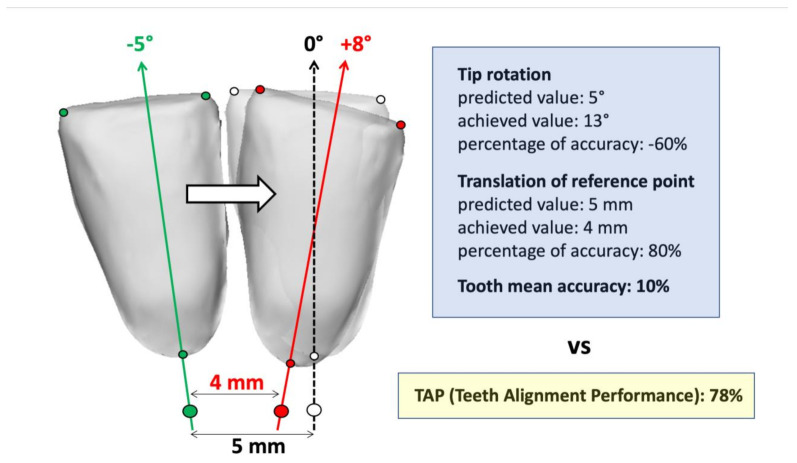
Illustrative, two-dimensional representation of a moving lower central incisor in its initial (pre-treatment, green landmarks), final (post-treatment, red landmarks), and predicted (dotted line, white landmarks) frontal position and orientation. Tip angle and the displacement of a translation reference point are simulated, and the corresponding percentage accuracies are calculated according to Kravitz and colleagues [13,14]; the resulting mean accuracy of the movement is finally compared to the TAP index.

The TAP is a normalized accuracy index that reports how close the final landmarks are to their expected final position, with respect to the linear distance they were planned to achieve, without the need to report and average each of the 6 DOF movement components [13,14] (Figure 3). A perfect achievement of the predicted movement would have a score of 100%.

A sensitivity threshold was set to ignore a target tooth from the analysis when the sum of its 3 landmark prescribed displacements was lower than 1.5 mm.

### 2.4. Statistical Analysis

To assess the reliability of the TAP index, that is, to what extent a landmark’s identification (operator bias) and tooth superimposition (surface matching bias) may affect its variability, 22 arches were selected to obtain appropriate sample sizes for both global arch and tooth type inter-examiner comparisons. Sample sizes of 20 were indicated to achieve a power of 80% and a two-sided level of significance of 5%, for detecting an effect size of 0.7 between pairs. However, for upper canines, an acceptable sample size was not attained, and their data were not reported.

Descriptive statistics of the expected displacement (Mi-Ex), the missed displacement (Mf-Ex), and the TAP index were calculated for each tooth type, together with the TDI, and each arch, separately for the two examiners’ measurements. Data from each patient’s right and left homologous teeth within each arch were pooled for upper and lower central incisors, lateral incisors, and canines. Median and interquartile range (IQR) were calculated in place of mean and standard deviation (SD) when samples were not normally distributed according to the Shapiro–Wilk test.

To test the inter-examiner reproducibility of the dependent variables, Student’s paired t-test was applied to check for the presence of systematic differences between the two independent observations; Dahlberg’s Random Error of Measurement (REM) and Intra-class Correlation Coefficient (ICC) were calculated to quantify the mean random variation of the recording and the inter-examiner correlation, respectively.

Correlations among arches’ Mi-Ex displacement, TAP index, and absolute value of inter-rater TAP discrepancy were also assessed by means of Spearman’s correlation coefficient for non-normally distributed samples.

For all inferential tests, *p* < 0.05 was considered statistically significant.

## 3. Results

Overall, the data samples were normally distributed, except for the absolute value of inter-rater TAP discrepancy, Mi-Ex, and Mf-Ex variables.

Table 1 and Table 2 report the descriptive and reliability statistics related to the entire arches and single target teeth, respectively. Values of upper canines are not reported due to their insufficient sample size (*n* = 2).

The TAP of the arches ranged from 1% to 79%, while their overall expected displacement varied between 3.4 mm and 45.9 mm.

Among the single teeth, on average, the displacement of lower lateral incisors occurred with the highest accuracy, while the displacement of lower central incisors had the lowest performance (TAP of 61% and 45%, respectively). The median values of the expected displacements (sum of the 3 landmark expected displacements) were 3.7 mm for upper central and lateral incisors, 3.4 mm for lower central incisor, 4.1 mm for lower lateral incisor, and 2.5 mm for lower canine.

**Figure 4:**
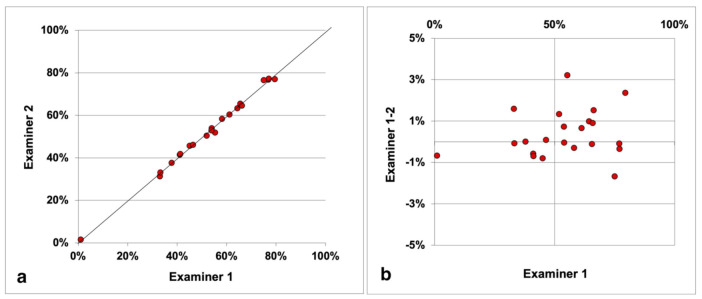
Scatter plot with the identity line (**a**) and Bland-Altman plot (**b**) showing the comparison between the TAP (Teeth Alignment Performance) values provided by the two examiners from the 22 analyzed arches.

There was no systematic inter-examiner discrepancy associated with the TAP calculation. Mandibular canines showed the highest REM (3%) and the lowest ICC (0.95) of the TAP index, but they were also the teeth with the smallest Mi-Ex (median, 2.5 mm). All the other intraclass correlation coefficients were close to one, including the one associated to the global arch TAP (Figure 4), whose random error of measurement was 1%, indicating excellent repeatability.

The only significant systematic difference between the two examiners was observed in the TDI of the upper central incisors (means of 28.1 mm vs. 28.3 mm, *p* < 0.001), whose REM was the lowest (0.2 mm), while the highest REM was reported for the TDI of the mandibular central incisors (0.8 mm).

No significant Spearman’s correlation coefficient was found between arches’ TAP and expected displacement (rs = 0.267, *p* = 0.230), TAP, and absolute value of inter-rater TAP discrepancy (rs = 0.240, *p* = 0.282), as well as between absolute value of inter-rater TAP discrepancy and expected displacement (rs = 0.216, *p* = 0.335).

## 4. Discussion

The present study described and tested the reproducibility of a novel accuracy index, the Teeth Alignment Performance index (TAP), devised to quantify the fulfillment rate of an orthodontic treatment in a single, comprehensive parameter. A non-homogeneous sample was selected in order to evaluate the reliability of the proposed method in different clinical conditions and recording procedures.

When evaluating the outcomes of an orthodontic treatment, a system of concomitant factors plays a role in determining efficient teeth movement: plaster/virtual model precision, type of teeth movement, teeth movement sequencing and staging, removable/fixed appliance design and material, crown and root morphology of the teeth, bone density, and certain systemic conditions [15]. In addition, the clinician’s accuracy in placing attachments and performing IPR, as well as the patient’s compliance and motivation, have a crucial effect on the final performance [5,14]. The TAP index assesses the performance of a multi-factorial system composed of alignment device, digital treatment plan, clinician, and patient. This index does not quantify how much the teeth are finally aligned and leveled; it is a normalized accuracy indicator reporting the fulfillment rate of the planned teeth movement, and its score depends on all the factors listed above.

The development of digital systems and efficient surface-to-surface matching algorithms have been previously used by some authors to segment the teeth crowns before the treatment and superimpose them to the post-treatment predicted and achieved virtual models, obtaining a transformation matrix describing the roto-translation that occurred [2,12,13,14,16,17]. However, these studies reported values of teeth rotation and/or translation without describing the landmarks used to create each tooth reference system, which makes it impossible to replicate their calculations for a direct comparison with the TAP score. In addition, when the corresponding percentage accuracy indices are reported [13,14], their calculations are liable to provide negative values (Figure 3) or be unapplicable due to mathematical singularity (when the predicted value of a movement component is null, but the orthodontic device induces its change). In addition, when attempting to provide an average accuracy index, the authors pooled together the mean values of movement accuracies obtained from angles and distances, which would be misleading to interpret as an overall movement accuracy; for example, when the planned tooth movement is mainly a translation to close an important gap, a small concomitant rotation that is only partially achieved would have an excessive detrimental impact on the overall mean percentage accuracy (Figure 3).

The movement of a three-dimensional rigid body can be described, uniquely determining the positions of at least three unaligned points. The three landmarks set by this method for each anterior tooth crown were chosen to be easily detectable in all conditions, even with crowding. Furthermore, they make the TAP sensitive to all the three rotational components (tip, torque, rotation), as well as the three translational axial components. Among the main problems associated with orthodontic treatments, especially when clear aligners are used, are both the uncorrected rotation and unwanted tip/torque of the teeth [9,18], which an effective accuracy index must account for (Figure 3). The TAP score is also sensitive to possible loss of anchorage of adjacent teeth, since their unprescribed movement is necessarily accompanied by a limited movement of the target teeth.

The sensitivity threshold of the predicted displacement of a target tooth was set to consider only clinically relevant movements [2,17] and to account for the bias in models’ superimposition [19].

No systematic inter-examiner discrepancy of the TAP index was observed, not even when a slight systematic inter-rater difference in landmark labelling was detected, as for the upper central incisors. In addition, all ICC values (>0.95) showed excellent inter-rater agreement, and the small REM, ranging from 1% for the arch TAP to 3% for the lower canine TAP, indicates that this accuracy index is highly reliable.

Moreover, the absence of linear correlations between the absolute value of inter-rater TAP discrepancy and both the TAP score and the overall expected displacement demonstrates robustness and consistency of the TAP index; even in the treatment case in which the smallest overall teeth movement was expected (Mi-Ex, 3.4 mm) and almost none occurred, the two independent TAP scores reported by the two examiners were 1% and 2%.

Besides the novel accuracy index, to the best of our knowledge, this is the first time that clinical outcomes of orthodontic teeth movement treated with directly printed (instead of thermoformed) clear aligners have been reported. Despite being an emerging technology in this orthodontic field [20], its performance results seem promising, and further studies will assess its evolution, comparing its accuracy scores with the ones obtained from traditional techniques. For now, the lack of linear correlation found between the TAP index and the overall expected displacement suggests that the case complexity of anterior teeth movement has no influence on treatment performance, in line with what has been observed using the well-known Invisalign^®^ system [13].

An important advantage of the presented method is that no specific reference system has to be established, neither a global one for the arch, nor local ones for each tooth, since it is based on linear displacements of the landmarks. However, the superimposition of the three digital models (pre-treatment, post-treatment, and expected) is necessary and represents a critical aspect of this kind of performance analysis. The overlap led by means of the untreated, posterior teeth is a largely used method [9,13,15,16,19,21], relying on the assumption that they remain stable throughout an orthodontic treatment involving the anterior teeth. However, acting as anchoring teeth, the untreated posterior teeth generally move, even if minimally, in response to the action–reaction of Newton’s third law of motion. In addition, when clear aligners are worn, a common side effect is the unwanted intrusion of the molars due to the material thickness interposed occlusally [4,22], which causes the anterior teeth of the post-treatment model to appear slightly intruded after the superimposition. Alternatively, model overlap can be obtained by the best-fit matching of the entire arch [2,14,17]; however, with this method the target and stationary teeth are mixed up. Palatal rugae [23,24] and palatal vault [22,25] would be reliable regions to drive the arches’ superimposition, but they are applicable with maxillary treatments only. A fourth method of model superimposition makes use of cone-beam computed tomography scans (CBCT), taken both at the beginning and at the end of the treatment [25]. This technique allows for the evaluation of posterior teeth movement, but it would not be ethically justified, because it would expose the patients to ionizing radiation without any diagnostic or therapeutic benefit.

For the purposes of this study, we opted for the superimposition of the models on the untreated posterior teeth, since no pre- and post-treatment CBCT were available, and only the six upper and lower frontal teeth were planned to move, and then we investigated. This constitutes a limitation of this study, which was not able to provide reliability data associated to the upper canines, due to the low sample size. However, the TAP repeatability reported in the present study and its applicability are not affected by the technique chosen for the superimposition of the arches; its use can be reasonably extended to the posterior teeth when their movement is also digitally planned, provided that model overlap is not led by the posterior teeth in that case.

## 5. Conclusions

The proposed TAP index was proven to reliably assess the performance of teeth alignment according to a digital plan, with the net of the system of concomitant factors affecting its attainment. Its use is indicated both as a research tool, to compare different orthodontic devices and strategies, and as a clinical tool, providing clinicians with quantitative data to support their evaluation of the progression of a treatment and gain experience in the optimal handling of an orthodontic device. The proposed method is also suitable to be implemented in the clinical digital workflow.

## Figures and Tables

**Table 1:** Descriptive and reliability statistics of the expected displacement (Mi-Ex), missed displacement (Mf-Ex), and TAP index of the 22 arches assessed.

	Mi-Ex (mm)	Mf-Ex (mm)	TAP (%)
Mean/*Median*	*13.7–13.6*	*5.1–5.2*	54–53
SD/*IQR*	*7.5–7.3*	*5.1–5.0*	18–18
Mean of differences	0.0	0.0	0
SD of differences	0.4	0.2	1
*p* (paired t-test)	0.833	0.239	0.141
REM	0.3	0.1	1
ICC	1.00	1.00	1.00

SD, standard deviation; IQR, inter-quartile range; REM, random error of measurement; ICC, intraclass correlation coefficient. For median and IQR (italics), the values yielded by the two examiners are reported whereas mean and SD (lowercase block) are reported only for TAP score.

**Table 2:** Descriptive and reliability statistics of the TAP index associated to the anterior teeth.

	Upper Central Incisor	Upper Lateral Incisor	Lower Central Incisor	Lower Lateral Incisor	Lower Canine
*n*	20	19	14	19	9
Mean (%)	48–48	56–55	45–44	61–61	55–55
SD (%)	26–26	16–16	17–17	18–19	14–12
Mean of differences (%)	0	1	1	0	0
SD of differences (%)	1	2	2	2	5
*p* (paired t-test)	0.558	0.139	0.344	0.970	0.875
REM (%)	1	2	2	2	3
ICC	1.00	0.99	0.99	0.99	0.95

SD, standard deviation; REM, random error of measurement; ICC, intraclass correlation coefficient. For mean and SD, the values yielded by the two examiners are reported. Values of upper canines are not shown due to insufficient sample size.

## Data Availability

The data presented in this study are available on request from the corresponding author. The data are not publicly available due to privacy limitations.

## References

[1] Rossini G., Parrini S., Castroflorio T., Deregibus A., Debernardi C.L. (2016). Diagnostic accuracy and measurement sensitivity of digital models for orthodontic purposes: A systematic review. Am. J. Orthod. Dentofac. Orthop..

[2] Larson B.E., Vaubel C.J., Grünheid T. (2012). Effectiveness of computer-assisted orthodontic treatment technology to achieve predicted outcomes. Angle Orthod..

[3] Onyeaso C.O., Begole E.A. (2007). Relationship between index of complexity, outcome and need, dental aesthetic index, peer assessment rating index, and American Board of Orthodontics objective grading system. Am. J. Orthod. Dentofac. Orthop..

[4] Buschang P.H., Ross M., Shaw S.G., Crosby D., Campbell P.M. (2014). Predicted and actual end-of-treatment occlusion produced with aligner therapy. Angle Orthod..

[5] Gu J., Tang J.S., Skulski B., Fields H.W., Beck F.M., Firestone A.R., Kim D.-G., Deguchi T. (2017). Evaluation of Invisalign treatment effectiveness and efficiency compared with conventional fixed appliances using the Peer Assessment Rating index. Am. J. Orthod. Dentofac. Orthop..

[6] Krieger E., Seiferth J., Marinello I., Jung B.A., Wriedt S., Jacobs C., Wehrbein H. (2012). Invisalign^®^ treatment in the anterior region: Were the predicted tooth movements achieved?. J. Orofac. Orthop..

[7] Ercoli F., Tepedino M., Parziale V., Luzi C. (2014). A comparative study of two different clear aligner systems. Prog. Orthod..

[8] Little R.M. (1975). The Irregularity Index: A quantitative score of mandibular anterior alignment. Am. J. Orthod..

[9] Drake C.T., McGorray S.P., Dolce C., Nair M., Wheeler T.T. (2012). Orthodontic tooth movement with clear aligners. ISRN Dent..

[10] Macauley D., Garvey T.M., Dowling A.H., Fleming G.J. (2012). Using Littles Irregularity Index in orthodontics: Outdated and inaccurate?. J. Dent..

[11] Burns A., Dowling A.H., Garvey T.M., Fleming G.J. (2014). The reliability of Littles Irregularity Index for the upper dental arch using three dimensional (3D) digital models. J. Dent..

[12] Keilig L., Piesche K., Jager A., Bourauel C. (2003). Applications of Surface–Surface Matching Algorithms for Determination of Orthodontic Tooth Movements. Comput. Methods Biomech. Biomed. Eng..

[13] Kravitz N.D., Kusnoto B., BeGole E., Obrez A., Agran B. (2009). How well does Invisalign work? A prospective clinical study evaluating the efficacy of tooth movement with Invisalign. Am. J. Orthod. Dentofac. Orthop..

[14] Haouili N., Kravitz N.D., Vaid N.R., Ferguson D.J., Makki L. (2020). Has Invisalign improved? A prospective follow-up study on the efficacy of tooth movement with Invisalign. Am. J. Orthod. Dentofac. Orthop..

[15] Chisari J.R., McGorray S.P., Nair M., Wheeler T.T. (2014). Variables affecting orthodontic tooth movement with clear aligners. Am. J. Orthod. Dentofac. Orthop..

[16] Simon M., Keilig L., Schwarze J., Jung B.A., Bourauel C. (2014). Treatment outcome and efficacy of an aligner technique--regarding incisor torque, premolar derotation and molar distalization. BMC Oral Health.

[17] Grünheid T., Loh C., Larson B.E. (2017). How accurate is Invisalign in nonextraction cases? Are predicted tooth positions achieved?. Angle Orthod.

[18] Zhang X.-J., He L., Guo H.-M., Tian J., Bai Y.-X., Li S. (2015). Integrated three-dimensional digital assessment of accuracy of anterior tooth movement using clear aligners. Korean J. Orthod..

[19] Kravitz N.D., Kusnoto B., Agran B., Viana G. (2008). Influence of Attachments and Interproximal Reduction on the Accuracy of Canine Rotation with Invisalign. Angle Orthod..

[20] Tartaglia G., Mapelli A., Maspero C., Santaniello T., Serafin M., Farronato M., Caprioglio A. (2021). Direct 3D Printing of Clear Orthodontic Aligners: Current State and Future Possibilities. Materials.

[21] Charalampakis O., Iliadi A., Ueno H., Oliver D.R., Kim K.B. (2018). Accuracy of clear aligners: A retrospective study of patients who needed refinement. Am. J. Orthod. Dentofac. Orthop..

[22] Dai F.-F., Xu T.-M., Shu G. (2019). Comparison of achieved and predicted tooth movement of maxillary first molars and central incisors: First premolar extraction treatment with Invisalign. Angle Orthod..

[23] Ali B., Shaikh A., Fida M. (2016). Stability of Palatal Rugae as a Forensic Marker in Orthodontically Treated Cases. J. Forensic Sci..

[24] Zhou N., Guo J. (2019). Efficiency of Upper Arch Expansion with the Invisalign System. Angle Orthod..

[25] Pan Y., Wang X., Dai F., Chen G., Xu T. (2020). Accuracy and reliability of maxillary digital model (MDM) superimposition in evaluating teeth movement in adults compared with CBCT maxillary superimposition. Sci. Rep..

